# Biochar Addition Inhibits Nitrification by Shifting Community Structure of Ammonia-Oxidizing Microorganisms in Salt-Affected Irrigation-Silting Soil

**DOI:** 10.3390/microorganisms10020436

**Published:** 2022-02-14

**Authors:** Rong-Jiang Yao, Hong-Qiang Li, Jing-Song Yang, Xiang-Ping Wang, Wen-Ping Xie, Xing Zhang

**Affiliations:** 1 State Key Laboratory of Soil and Sustainable Agriculture, Institute of Soil Science, Chinese Academy of Sciences, Nanjing 210008, China; rjyao@issas.ac.cn (R.-J.Y.); lihongqiang@issas.ac.cn (H.-Q.L.); xpwang@issas.ac.cn (X.-P.W.); wpxie@issas.ac.cn (W.-P.X.); xzhang@issas.ac.cn (X.Z.); 2 College of Resources and Environment, University of Chinese Academy of Sciences, Beijing 100049, China

**Keywords:** community structure, ammonia-oxidizing microorganisms, nitrification, biochar, salt-affected soil

## Abstract

Biochar has been widely recognized as an effective and eco-friendly ameliorant for saline soils, but information about the mechanism of how biochar influences nitrification in salt-affected agroecosystem remains fragmented. An incubation experiment was performed on the salt-affected soil collected from a three-consecutive-year experiment at biochar application gradients of 7.5 t⋅ha^−1^, 15 t⋅ha^−1^ and 30⋅t ha^−1^ and under nitrogen (N) fertilization. Responses of the nitrification rate (NR), numbers of ammonia monooxygenase (*amoA*) gene copies, and community structures of ammonia-oxidizing bacteria (AOB) and archaea (AOA) to biochar application were investigated. The results indicated that, under N fertilization, the NR and numbers of *amoA*-AOB and *amoA*-AOA gene copies negatively responded to biochar addition. Biochar application increased the community diversity of AOB but decreased that of AOA. Biochar addition and N fertilization shifted the AOB community from *Nitrosospira*-dominated to *Nitrosospira* and *Nitrosomonas-dominated*, and altered the AOA community from *Nitrososphaera*-dominated to *Nitrososphaera* and *Nitrosopumilus*-dominated. The relative abundance of *Nitrosospira*, *Nitrosomonas* and *Nitrosopumilus* decreased, and that of *Nitrosovibrio* and *Nitrososphaera* increased with biochar application rate. Soil SOC, pH and NO_3_^−^-N explained 87.1% of the variation in the AOB community, and 78.1% of the variation in the AOA community was explanatory by soil pH and SOC. The SOC and NO_3_^−^-N influenced NR through *Nitrosovibrio*, *Nitrosomonas*, *Norank_c_environmental_samples_p_Crenarchaeota* and *amoA*-AOB and *amoA*-AOA gene abundance. Therefore, biochar addition inhibited nitrification in salt-affected irrigation-silting soil by shifting the community structures of AOB and AOA and reducing the relative abundance of dominant functional ammonia-oxidizers, such as *Nitrosospira*, *Nitrosomonas* and *Nitrosopumilus*.

## 1. Introduction

Nitrogen is an indispensable nutrient element for sustaining ecosystem productivity and plays a pivotal role in closing crop yield gaps to meet ever-increasing food demands [1]. In salt-affected areas worldwide, extensively distributed saline soils, which are promising reserve land resources for compensating for the shortfall in food requirements, exert adverse impacts on crop growth and nitrogen nutrient uptake and result in nitrogen loss and environmental problems such as greenhouse gas emissions and nonpoint source pollution [2]. The migration and transformation processes of nitrogen in agricultural ecosystems are greatly affected by soil salinity and derivative obstacle factors [3]. Therefore, the amendment of soil salinization hazards is indispensable for enhancing soil productivity, improving nitrogen nutrient utilization, and minimizing environmental losses of nitrogen [4].

Numerous measures have been developed to amend soil salinization by physically improving soil porosity, chemically accelerating ion exchange and leaching, and biologically promoting soil biochemical function [5,6]. Among these methods, biochar is globally recognized as an environmentally friendly amendment owing to merits such as easy accessibility of raw materials and its harmless and pollution-free nature [7] and has been widely used in saline soil amelioration [8]. To date, many efforts have been devoted to investigating the responses of nitrogen morphological conversion, most often through microbe-mediated processes, to biochar addition in saline environments. Therefore, the effect and mechanism of biochar addition on nitrification have received increasing attention, as nitrification is closely related to mineralization, fixation, and denitrification [9]. However, recent reviews of the effect of biochar on nitrification in salt-affected soil are inconsistent and even contradictory. The biochar effect on nitrification varies owing to different soil salinity levels, ionic compositions of soluble salts, soil textures, and initial nutrient statuses [10]. Most researchers have reported a stimulating effect of biochar on nitrification, as biochar promotes soil microbial activity, nutrient availability, and biochemical function due to its high specific surface area, hydrophilicity, and adsorption capacity [11]. Some researchers have also discovered an inhibitory effect of biochar on nitrification due to surface free radicals, increasing the soil pH, and reducing the bioavailability of NH_4_^+^-N [12,13].

Ammonia oxidation, catalyzed by ammonia-oxidizing bacteria (AOB) and ammonia-oxidizing archaea (AOA), is the rate-limiting step of autotrophic nitrification [14]. Previous reports have shown that AOB regulate ammonia oxidation in neutral and alkaline environments, whereas AOA dominate ammonia oxidation in low-pH environments [15]. In salt-affected soil, soil salinity has strong negative effects on the numbers of *amoA*-AOB and *amoA*-AOA gene copies, but the effects of biochar on the abundance of AOB and AOA in saline soil are inconsistent. The abundance of AOB and AOA decreased with biochar addition in saline-alkali soil, and AOB were more susceptible than AOA to biochar addition [16]. It was reported that biochar application significantly increased the abundance of AOB and AOA, and the rate and dynamics of nitrification were closely associated with the rate of biochar application [17]. In addition to the abundance, the community structures of AOB and AOA are altered by biochar addition in salt-affected soil, and the dominant genera *Nitrosospira*, *Nitrosomonas*, *Nitrosovibrio*, *Nitrososphaera* and *Nitrosopumilus* are the most frequently reported taxa that were sensitive to biochar addition [18]. It was also reported that biochar addition increased the diversity and abundance of *amoA*-AOB gene and shifted the AOB community structure from *Nitrosospira*-dominated toward *Nitrosomonas-*dominated [19]. It was in line with Shi et al. who revealed that biochar application enhanced the abundance of *amoA*-AOB and *amoA*-AOA genes, and the genera *Nitrosospira* (AOB) and *Nitrososphaera* (AOA) achieved absolute superiority [20].

In summary, the influencing mechanism of biochar application on the potential nitrification rate (PNR), abundance and community structure of ammonia-oxidizing microorganisms has attracted increasing interest. However, most of the present conclusions concerning salt-affected soils are derived from experiments conducted under certain conditions and are fragmented. Little is known about the mechanism of the functional and structural responses of nitrification to biochar addition in salt-affected irrigation-silting soil. In the present study, an aerobic incubation experiment was conducted on soils sampled from a three-consecutive-year field plot trial at different biochar application rates under N fertilization. The nitrification rate and the *amoA* gene copies, diversity and community structures of AOB and AOA were measured using quantitative PCR amplification and Illumina MiSeq sequencing. The primary objectives were to: (i) investigate the responses of the nitrification rate and numbers of *amoA*-AOB and *amoA*-AOA gene copies to biochar at different application rates; (ii) clarify the effect of biochar addition on the diversity, community ordinations and structures of AOB and AOA; and (iii) identify the causal relationships among soil properties, the numbers of *amoA*-AOB and *amoA*-AOA gene copies, relative abundance of dominant genera, and nitrification rate.

## 2. Materials and Methods

### 2.1. Description of Field Plot Trial

Soil samples used in the present experiment were collected from a three-consecutive-year field plot trial conducted in salt-affected farmland in Dengni Village (40°49.4′~40°49.8′ N, 106°54.7′~120°55.2′ E), Hanggin Rear Banner of Inner Mongolia, China. The field trial is located in the northwestern of Hetao Irrigation District (HID), which is a typical irrigated area in the upper and middle reaches of the Yellow River basin in China [21]. Details of the experimental site and field plot trial were given in [22]. Briefly, the field plot trial in a completely randomized plot design consisted of five treatments: CK (control, no N fertilization or biochar input), N (N fertilization at 225 kg N ha^−1^⋅yr^−1^), NB1 (N fertilization at 225 kg N ha^−1^⋅yr^−1^, biochar rate at 7.5 t ha^−1^), NB2 (N fertilization at 225 kg N ha^−1^⋅yr^−1^, biochar rate at 15 t ha^−1^) and NB3 (N fertilization at 225 kg N ha^−1^⋅yr^−1^, biochar rate at 30 t ha^−1^). Using the conventional irrigation and agronomic management practices, the sunflower (*Helianthus annuus* L.) variety “SH361” was planted in the field plot trial throughout the experimental period, i.e., from May 2017 to September 2019.

The biochar used in the field plot trial was produced by pyrolyzing and charring wheat straw at 400~450 °C for 4 h under oxygen-restricted circumstances. The basic properties of the biochar were detailed described in [23]. Biochar was added to field plots in May 2017 and manually mixed with 0−20 cm soil layer prior to the start of the experiment. Nitrogen fertilizer was applied to field plots in batches during the growth period of sunflower. The experiment lasted three consecutive years, and soil-biochar mixture samples at the 0−20 cm layer were obtained at the end of the experiment, i.e., after the sunflower harvest in September 2019. The fresh samples were air dried, crushed, sieved and mixed together to form one representative soil sample for each treatment. Each soil sample was subdivided into two subsamples: one was used for the lab analysis of basic soil properties, and the other was stored at 4 °C for the incubation experiment.

### 2.2. Soil Microcosm Construction and Incubation

Prior to the incubation experiment, soil microcosms were established for each treatment by adding 30 g of soil or a soil-biochar mixture (on an oven-dried basis) to a 250 mL Mason jar. All jars were moistened to 60% water-filled pore space (WFPS) using distilled water and placed in a thermostatic incubator for pre-incubation at 25 ± 1 °C in the dark for 3 days to revive soil microbial activity. The top of each jar was wrapped using plastic films with small holes to ventilate and prevent moisture losses. After pre-incubation, soil samples were collected as the initial soil status. Meanwhile, the soil microcosms were fertilized with (NH_4_)_2_SO_4_ solution at a rate of 200 mg N kg^−1^ dry weight soil and moistened to keep the soil moisture at 65% WFPS. Using the weighing method, deionized water was added to each microcosm to maintain constant moisture content during the incubation. The incubation lasted 35 days, and soil samples were collected from the microcosms on the 1st, 3rd, 7th, 10th, 15th, 25th and 35th days. Each treatment had 24 replicate microcosms and triplicate microcosms were used for each sampling, and 24 replicas were used up after eight soil samplings (including soil sampling after pre-incubation). The collected soil samples were sieved through a mesh size of 2 mm and subdivided into two subsamples: one was stored at 4 °C for soil chemical analysis, and the other was stored at −80 °C for soil microbiological analysis.

### 2.3. Laboratory Analysis

For the soil samples used in microcosm construction and incubation, the analyzed physio-chemical properties consisted of soil salinity (EC_1:5_), pH, cation exchange capacity (CEC), soil organic carbon (SOC), total nitrogen (TN), NH_4_^+^-N and NO_3_^−^-N contents, and available potassium (AP). The EC_1:5_ and pH were measured on 1:5 soil:water (*w*/*v*) suspensions. The CEC was measured using the ammonium acetate extraction method. The SOC and TN were analyzed by wet digestion with H_2_SO_4_-K_2_Cr_2_O_7_ and semimicro Kjeldahl digestion. Soil NH_4_^+^-N and NO_3_^−^-N contents were determined on a 1:5 soil:KCl (2 M) extract using ultraviolet spectrophotometry. The AP (Olsen P) was analyzed by the sodium bicarbonate extraction and colorimetric analysis. Detailed analytical procedures for the above soil attributes referred to [24]. The measured basic soil properties for all treatments are shown in Table 1.

For the incubation soil samples, concentrations of NH_4_^+^-N and NO_3_^−^-N were analyzed for all the samples collected on different dates, and the soil samples collected on the 10th day of incubation were selected for analyzing the ammonia monooxygenase (subunit A, *amoA*) gene copies and community structures of ammonia oxidizing bacteria (AOB) and ammonia-oxidizing archaea (AOA). This was done considering the dynamic characteristics of nitrification in salt-affected soils, and the differences in nitrification rate and *amoA* gene number were significant among different treatments on the 10th day.

### 2.4. Soil DNA Extraction, Quantitative PCR (qPCR) Amplification of amoA Genes

Soil total genomic DNA was extracted using the E.Z.N.A.^®^ Soil DNA Kit (Omega BioTek, Norcross, GA, USA) according to the procedure suggested by [25]. Using a NanoDrop^®^ ND-2000c UV-Vis spectrophotometer (NanoDrop Technologies, Wilmington, DE, USA), the content and purity of the extracted genomic DNA was quantified using agarose gel electrophoresis (AGE) at 1% content. Quantitative PCR (*q*PCR) was performed with duplicate sets of extracted DNA in an Mx3005P instrument (Stratagene, Santa Clara, CA, USA) with a Brilliant II SYBR Green QPCR Master Mix (Stratagene, La Jolla, CA, USA). The amplification primers, sequences and reaction conditions of *qPCR* for *amoA*-AOB and *amoA*-AOA genes are shown in Table S1. Detailed procedures of *q*PCR reaction, standard DNA preparation and count of gene copies for AOB and AOA were given in [22,26]. The *q*PCR amplification efficiencies were 98.32% and 97.55% for AOB and AOA, respectively.

### 2.5. Illumina MiSeq Sequencing and Phylogenetic Analysis

The above obtained PCR products were checked using 2% agarose gel electrophoresis and purified using an AxyPrep DNA gel extraction kit (Axygen, Union City, CA, USA) to remove any unspecified products. Then, the PCR products were eluted with Tris-HCl buffer and checked using 2% agarose gel electrophoresis again. Purified amplicons were mixed in equimolar amounts and sequenced on an Illumina MiSeq Benchtop Sequencer (Illumina, San Diego, CA, USA). After sequencing, the sequences were checked and optimized using the Trimmomatic software. Operational taxonomic units (OTUs) were defined according to 97% similarity using USEARCH v7 [27]. The representative sequences of the main OTUs were selected for alignment in the NCBI database to find the homologs and the closest sequences. The detailed procedure of bioinformatic analysis was described in [28].

### 2.6. Statistical Analysis

The nitrification rate (NR) was calculated according to the formulae proposed by [29]. The diversity and abundance-based richness within the community was evaluated using indices including observed OTUs, Chao1, ACE, Shannon and Simpson [30]. Based on SPSS Statistics 17.0 (IBM Company, Armonk, NY, USA), one-way analysis of variance (ANOVA) was used to compare the basic soil properties, nitrification rate, numbers of *amoA*-AOB and *amoA*-AOA gene copies, and community richness and diversity among different treatments. The relative abundance of dominant taxa at the order and genus levels was also compared using one-way ANOVA for the AOB and AOA, respectively. CANOCO 5.0 (Microcomputer Power, Ithaca, NY, USA) was used to perform principal coordinate analysis (PCoA) and redundancy analysis (RDA) to reveal the community ordinations and environmental relationships. Using AMOS 23.0 (IBM, Meadville, PA, USA), structural equation modeling (SEM) was conducted to identify the causal effects of soil microhabitat traits, relative abundance of dominant genera of AOB and AOA, and the numbers of *amoA*-AOB and *amoA*-AOA gene copies on nitrification rates under biochar addition. The criteria for assessing the performance of model fitting referred to [31].

## 3. Results

### 3.1. Nitrification Rate and amoA Gene Number

The nitrification rate (NR) within the 7th and 10th day of incubation and the numbers of *amoA*-AOB and *amoA*-AOA gene copies are given in Table 2. The average net NR values were 28.15, 27.64, 21.8, 15.74 and 11.67 mg⋅kg^−1^⋅d^−1^ for the CK, N, NB1, NB2 and NB3 treatments, respectively. One-way ANOVA results showed that the NR value under the CK treatment was not different from that under the N treatment, and the NR values under the CK and N treatments were significantly higher than those under the NB2 and NB3 treatments. Overall, biochar addition resulted in the decrease of NR and the treatment with the highest amount of biochar (30 t⋅ha^−1^) had the lowest NR value. Actually, the NR varied temporally during the autotrophic nitrification process (Supplementary Figure S1), and the variation in NR could be ascribed to the dynamics of community structure and function of ammonia-oxidizing microorganisms, bioavailability of substrate, and soil microhabitat traits [23].

The measured average number of *amoA*-AOB gene copies was 8.21 ± 0.54, 4.61 ± 0.64, 3.52 ± 0.63, 2.97 ± 0.81 and 2.50 ± 0.43 × 10^7^ g^−1^ soil under the CK, N, NB1, NB2, and NB3 treatments, respectively (Table 2), whereas the value of *amoA*-AOA was 3.71 ± 0.49, 2.12 ± 0.20, 1.83 ± 0.36, 0.96 ± 0.15, and 0.71 ± 0.10 × 10^6^ g^−1^ soil, respectively. The *amoA*-AOB gene copies outnumbered *amoA*-AOA gene copies for all the treatment, indicating that *amoA*-AOB was predominant in terms of *amoA* gene abundance. Following the N and NB1 treatments, the CK treatment had the highest number of *amoA-*AOB gene copies among all the treatments, and the *amoA*-AOB gene copies under the N treatment were significantly higher than those under the NB2 and NB3 treatments. This was also observed for the *amoA*-AOA gene copies, i.e., the NB2 and NB3 treatments had the lowest numbers of *amoA-*AOA gene copies. Overall, the abundance of both *amoA*-AOB and *amoA*-AOA genes was negatively responsive to biochar addition.

### 3.2. Richness and Diversity of AOB and AOA Communities

The diversity indices of AOB and AOA calculated from 16S rRNA gene amplicon sequencing are shown in Table 3. The N treatment increased the OTUs of AOB by 44.78% in comparison with the CK treatment. Compared with the N treatment, the NB1, NB2 and NB3 treatments decreased the OTUs of AOB by 3.09%, 5.16% and 9.28%, respectively. A decreasing trend was also observed for AOA under biochar addition. The Chao1 representing the community richness varied among different treatments and between AOB and AOA. N fertilization and biochar addition significantly increased the Chao1 index of AOB, and the NB3 treatment had the highest Chao1 index of 33.67. Nevertheless, no obvious difference was observed for the Chao1 index of AOA. The average ratios of OTUs/Chao1 were 95.42 ± 6.92% and 90.00 ± 6.37% for AOB and AOA, implying that the sequencing efforts for AOB were more exhaustive than those for AOA. Shannon and Simpson, the community diversity indices, showed clear responses to biochar addition for AOB. In comparison with the N treatment, NB1, NB2 and NB3 treatments increased the Shannon index but decreased the Simpson index of AOB. However, the Shannon and Simpson indices of AOA exhibited opposite responses to biochar addition. The coverage index was not responsive to N fertilization or biochar addition for either AOB or AOA.

### 3.3. Structural Characteristics of AOB and AOA Communities

Table 4 shows the relative abundance of the dominant taxa at the order level. For AOB, the community composition at the order rank was affiliated with three dominant orders: *Nitrosomonadales*, *Unclassified_k_norank_d_Bacteria*, and *Norank_p_ammonia_oxidizing_bacteria_ensemble*. *Nitrosomonadales* accounted for 95.44% of the total AOB community under the CK treatment, whereas the percentage declined to 81.59%, 73.26%, 63.08% and 58.87% under the N, NB1, NB2 and NB3 treatments, respectively. The relative abundance of *unclassified_k_norank_d_Bacteria* and *Norank_p_ammonia_oxidizing_bacteria_ensemble* increased with N fertilization and biochar addition. For AOA, the dominant taxa were *Nitrososphaerales*, *Nitrosopumilales*, *Norank_c_environmental_samples_p_Crenarchaeota* and *Unclassified_k_norank_d_Archaea* (Table 4). The relative abundance of *Nitrososphaerales* was 37.95% under the CK treatment, whereas the percentage increased to 43.15%, 58.76%, 60.23% and 51.02% under the N, NB1, NB2 and NB3 treatments, respectively. This ascending trend was also observed for *Unclassified_k_norank_d_Archaea*. *Nitrosopumilales* was negligible under the CK treatment, and N fertilization increased the percentage of *Nitrosopumilales* to 29.37%, but biochar addition decreased its relative abundance under N fertilization. Biochar addition also exerted a negative influence on the relative abundance of *Norank_c_environmental_samples_p_Crenarchaeota*.

**Figure 1:**
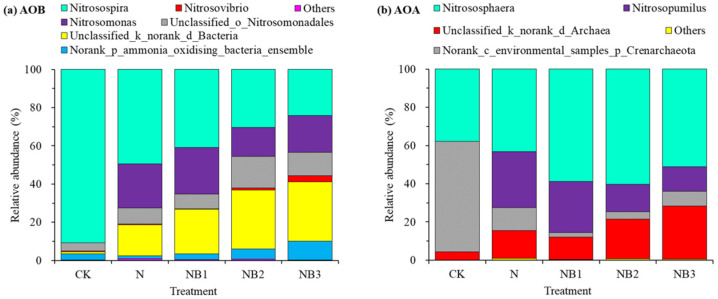
Relative abundance of AOB (**a**) and AOA (**b**) community structures at the genus level.

Figure 1a presents the relative abundance of the AOB community at the genus level. The most frequent genera (average relative abundance ≥ 5%) consisted of *Nitrosospira* (47.17 ± 24.97%), *Nitrosomonas* (16.36 ± 10.07%), *Unclassified_o_Nitrosomonadale*s (9.73 ± 6.01%) and *Unclassified_k_norank_d_Bacteria* (20.49 ± 12.85%). Genera *Norank_p_ammonia_oxidizing_bacteria_ensemble* (4.60 ± 4.38%) and *Nitrosovibrio* (1.09 ± 1.16%) belonged to a second group with a lower but still important percentage (1% ≤ average relative abundance < 5%). N fertilization significantly decreased the relative abundance of *Nitrosospira* but increased that of *Nitrosomonas* and *Unclassified_k_norank_d_Bacteria*. Furthermore, biochar addition increased the relative abundance of *Nitrosovibrio*, *Unclassified_k_norank_d_Bacteria* and *Norank_p_ammonia_oxidizing_bacteria_ensemble*, but decreased that of *Nitrosospira* under N fertilization. Genera *Nitrosomonas* and *Unclassified_o_Nitrosomonadales* showed no distinct response to biochar addition.

**Figure 2:**
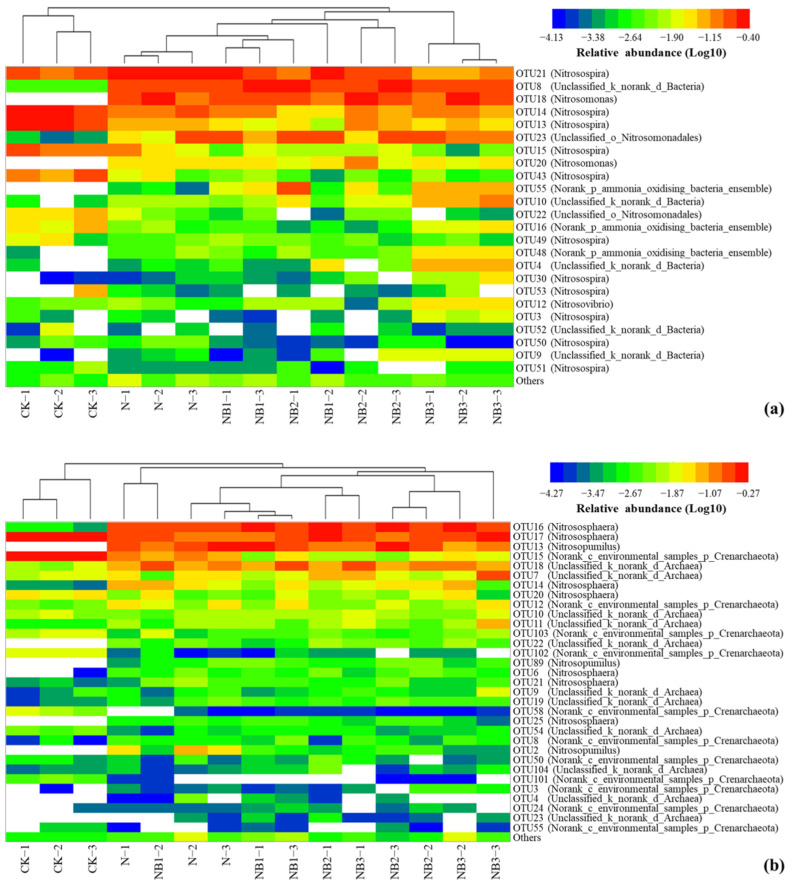
Heat maps showing taxa frequency distribution of OTUs for AOB (**a**) and AOA (**b**) at the genus level, plus the cluster structure across all the treatments.

The relative abundance of the AOA community at the genus level is shown in Figure 1b. The predominant taxa were *Nitrososphaera* (50.22 ± 11.27%), *Norank_c_environmental_samples_p_Crenarchaeota* (16.77 ± 21.49%), *Nitrosopumilus* (16.69 ± 13.88%) and *Unclassified_k_norank_d_Archaea* (15.78 ± 9.64%). The genus *Nitrosopumilus* was barely observed under the CK treatment. The N fertilization significantly enhanced the percentage of *Nitrosopumilus* and *Unclassified_k_norank_d_Archaea*, and decreased that of *Norank_c_environmental_samples_p_Crenarchaeota*. The further application of biochar, by contrast, decreased the relative abundance of *Nitrosopumilus* and *Norank_c_environmental_samples_p_Crenarchaeota*, whereas increased that of *Nitrososphaera* and *Unclassified_k_norank_d_Archaea*. Figure 2 presents the AOB and AOA community heatmap showing the frequency distribution of the dominant genera as well as the cluster structure.

### 3.4. AOB and AOA Community Ordinations

**Figure 3:**
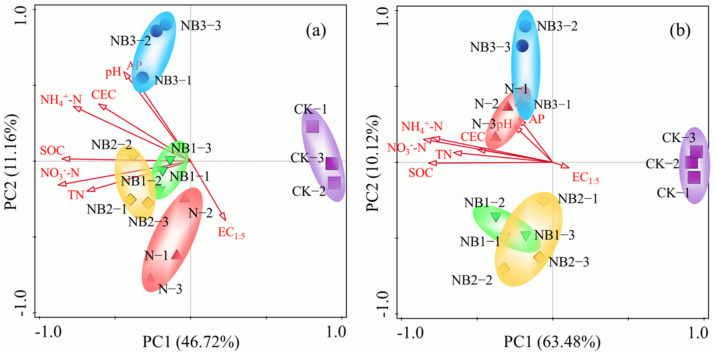
Principal coordinate analysis of AOA (**a**) and AOB (**b**) communities in soils with different salinities.

Principal coordinates analysis (PCoA) was used to evaluate the dissimilarity of the AOA and AOB communities. The obtained unconstrained ordination of the AOB and AOA communities and basic soil properties demonstrated that all treatments were clearly separated according to management practices (Figure 3). The first two PCs explained 57.88% of the variability in the AOB community and 73.60% of the variability in the AOA community. For AOB, the first principal component (PC1), which explained 46.72% of the variation in the data, separated the communities in the CK treatment from those in the other treatments. The second principal component (PC2) explained 11.16% of the data variance and separated the communities in the NB1, NB2 and NB3 treatments from those in the N treatment (Figure 3a). Likewise, AOA communities were also well separated, with PC1 explaining 63.48% of the variation and PC2 explaining 10.12% of the variance (Figure 3b). Overall, the two components clearly separated the AOB and AOA community compositions according to the differences in management practices, including N fertilization and biochar addition.

**Figure 4:**
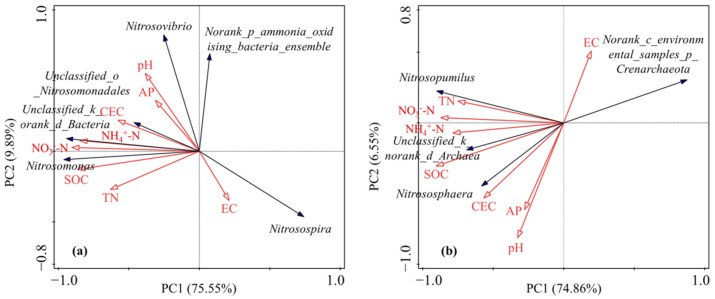
Redundancy analysis (RDA) diagram of the relationship between AOB (**a**) and AOA (**b**) community compositions (relative abundance of genus) and environmental factors.

The relationships between environmental variables and the community compositions of AOB and AOA were determined using redundancy analysis (RDA). Figure 4 shows the environment-species relevance based upon the relative abundance data matrix of dominant taxa at the genus level. Apparently, soil SOC, NO_3_^−^-N and pH were the significant environmental factors shaping the AOB community structure. The contribution of the environmental variable to the total explanatory variance is followed by the solely explanatory variance by this variable: SOC-61.8% (53.1%), pH-14.3% (12.3%), and NO_3_^−^-N-11.0% (9.4%). The first axis explained 69.90% of the variation (*p* < 0.01) and was correlated with soil NO_3_^−^-N and SOC, indicating that the first axis may characterize soil nutrient status. The second axis explained 4.52% of the variation (*p* < 0.05) and was correlated with soil pH, representing the status of the soil alkalinity. The differences in soil SOC, NO_3_^−^-N and pH, as induced by N fertilization and biochar addition, contributed to the structural variation of the AOB community. For AOA, the community structure was mainly dominated by SOC and pH, and the explanatory contribution and variance related to the environmental variables were SOC-67.5% (54.3%) and pH-10.6% (8.5%). The first axis explained 54.86% of the variation (*p* < 0.01) and characterized the soil nutrient status. The second axis represented the soil alkalinity status and explained 7.94% of the variation (*p* < 0.01). The structural variation of the AOA community could be mainly ascribed to the difference in soil SOC and pH.

### 3.5. Linking Basic Soil Properties, Dominant Taxa and amoA Gene Copies to NR Using SEM

The dependence between the relative abundance of dominant genera of AOB and AOA and nitrification rates, *amoA* gene copies and basic soil properties, as expressed by Spearman’s rank correlation coefficients, is given in Table 5. The NR exhibited a significantly positive correlation with the relative abundance of genera *Nitrosospira* and *Norank_c_environmental_samples_p_Crenarchaeota*, but a negative correlation with that of genera *Nitrosovibrio*, *Unclassified_k_norank_d_Bacteria*, *Norank_p_ammonia_oxidizing_bacteria_ensemble*, and *Unclassified_k_norank_d_Archaea*. Likewise, the numbers of both *amoA*-AOB and *amoA*-AOA gene copies showed significantly positive responses to the relative abundance of genera *Nitrosospira* and *Norank_c_environmental_samples_p_Crenarchaeota*, but negative responses to that of genera *Nitrosomonas*, *Unclassified_o_Nitrosomonadales*, *Nitrosovibrio*, *Unclassified_k_norank_d_Bacteria*, *Nitrososphaera*, and *Unclassified_k_norank_d_Archaea*. Interestingly, the relative abundance of genera *Nitrosospira* and *Norank_c_environmental_samples_p_Crenarchaeota* showed negative correlation with most basic soil properties, but that of genera *Unclassified_k_norank_d_Bacteria* and *Unclassified_k_norank_d_Archaea* showed positive correlation with most basic soil properties.

**Figure 5:**
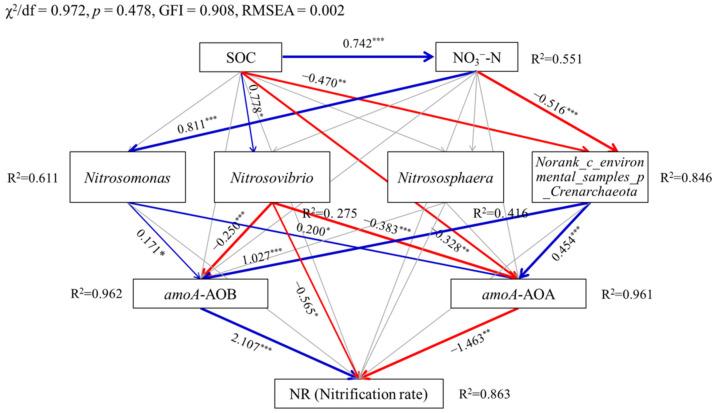
Structural equation models showing the effects of soil properties (SOC and NO_3_^−^-N) induced by biochar addition, dominant taxa of ammonia-oxidizing microorganisms, and *amoA* gene abundance on the soil nitrification rate (NR) at the genus level. Blue and red arrow lines indicate significant positive and negative relationships, respectively. Gray arrow lines indicate nonsignificant relationships. Numbers above arrow lines are standardized path coefficients. R^2^ indicates the proportion of variance explained by the model. *** *p* < 0.001; ** *p* < 0.01; * *p* < 0.05.

Figure 5 presents the relationships among the soil properties, the dominant genera of ammonia-oxidizing microorganisms, abundance of *amoA* gene, and nitrification rates using structural equation models (SEMs). The causality among the above attributes was accurately captured by the SEM from the criteria of model performance, i.e., *χ*^2^/*df* of 0.972, *p* of 0.478, *GFI* of 0.908, and RMSEA close to 0. SOC showed positive direct influences on the relative abundance of the genera *Nitrosovibrio* (*p* < 0.05) and NO_3_^−^-N (*p* < 0.001), but negative direct influences on the relative abundance of *Norank_c_environmental_samples_p_Crenarchaeota* (*p* < 0.01) and number of *amoA*-AOA gene copies (*p* < 0.01). Soil NO_3_^−^-N content had a positive direct influence on the relative abundance of *Nitrosomonas* (*p* < 0.001), and a negative direct influence on that of *Norank_c_environmental_samples_p_ Crenarchaeota* (*p* < 0.001). Additionally, the numbers of *amoA*-AOB and *amoA*-AOA gene copies were positively altered by the relative abundance of *Nitrosomonas* (*p* < 0.05) and *Norank_c_environmental_samples_p_ Crenarchaeota* (*p* < 0.001), but negatively altered by the relative abundance of *Nitrosovibrio* (*p* < 0.001). In addition to the direct influence, SOC had an indirect influence on NR through the numbers of *amoA*-AOB and *amoA*-AOA gene copies, which were directly responsive to the relative abundance of *Nitrosovibrio* (*p* < 0.001). Soil NO_3_^−^-N content also had an indirect positive influence on NR through the numbers of *amoA*-AOB and *amoA*-AOA gene copies, which were directly influenced by *Nitrosomonas* (*p* < 0.05). It was interesting to find that a common pathway through the relative abundance of *Norank_c_environmental_samples_p_Crenarchaeota* existed for SOC and NO_3_^−^-N content. That was, both SOC and NO_3_^−^-N content had indirect influence on NR through the relative abundance of *Norank_c_environmental_samples_p_Crenarchaeota*, and numbers of *amoA*-AOB and *amoA*-AOA gene copies. A total of 86.3% of the variation in NR was explained by the SEM model, and the proportions of the explainable variation in the relative abundance of *Nitrosomonas*, *Nitrosovibrio*, *Nitrososphaera*, and *Norank_c_environmental_ samples_p_Crenarchaeota*, and numbers of *amoA*-AOB and *amoA*-AOA gene copies were 61.1%, 27.5%, 41.6%, 84.6%, 96.2% and 96.1%, respectively.

## 4. Discussion

### 4.1. Responses of Soil Properties, Nitrification Ability, and amoA Gene Copies to Biochar Addition in Salt-Affected Soil

Biochar has been extensively used as an effective amendment for soil salinization hazards because it enhances the nutrient supply capacity, modulates porosity and pore size, improves hydraulic parameters, and promotes soil aggregate structure [32]. Usman et al. found that the inhibitory effect of soil salinization on vegetative growth tended to decline owing to the increases in soil organic matter and nutrient availability induced by the application of biochar, especially at high application rates [33]. This was in line with our study showing that biochar addition improved soil pH, CEC, SOC, NH_4_^+^-N and AP (Table 1). Zhu et al. discovered that biochar addition as a buried layer in the soil profile caused a breakdown in the continuity of capillary movement and accelerated the leaching rate of soluble salts because of the large pore volume and pore size of biochar [34]. However, this was not found in the present study, and the soil salinity was not different among treatments. The explanation was that excessive flooding irrigation, which exceeded the water requirements of crops and salt leaching, are commonly used in the Hetao Irrigation District. Moreover, biochar addition was found to improve nutrient supply capacity in saline soil owing to its high specific surface area and saturation moisture content. Yang et al. reported that the leaching loss of soil NO_3_^−^-N was reduced at any biochar application rate, and a high biochar application rate reduced soil NH_4_^+^-N leaching, but soil NH_4_^+^-N leaching significantly increased at a low biochar application rate (<1 wt%) [35]. This was also observed in non-saline and acidic soils [36,37], and coincided with the findings of this study.

Soil nitrification rate was closely associated with the abundance of *amoA* gene, and AOB-dominated nitrification and *amoA-*AOB gene abundance were predominant in alkaline soil, whereas AOA-dominated nitrification and *amoA*-AOA gene abundance were dominant in acidic soil [38]. Data on the influence of biochar addition on nitrification and *amoA* gene abundance in saline soils are currently inconsistent for different soil textures, salinization types, and nutrient statuses, i.e., promotion [17,39], inhibition [40,41], or indifference [42]. The possible stimulating pathways of biochar addition on nitrification in salt-affected soil include: (1) the improvement of biological activity of functional microorganisms associated with nitrification [11]; (2) the provision of favorable microhabitats for the growth of ammonia-oxidizing microorganisms by biochar owing to its high cation exchange capacity (CEC), hydrophilicity, and adsorption capacity [43]; and (3) the promotion of nitrogen mineralization and NH_4_^+^-N concentration, which acts as a substrate for nitrification [44]. In contrast, there are still many factors contributing to nitrification inhibition under biochar addition: (1) the increase of soil pH and ammonia volatilization, and the decline of the substrate content for nitrification [45]; (2) the reduction of the bioavailability of NH_4_^+^-N through physical adsorption and pore filling [46]; and (3) the shift of the community structure of functional microorganisms due to the reactive functional groups and free radicals on the biochar surface [12]. In fact, the performance of biochar addition largely depends on the prevailing factors in the nitrification process.

### 4.2. Biochar Addition Shifted the Community Structures of AOB and AOA in Salt-Affected Soil

The rate-limiting step of nitrification, i.e., ammonia oxidation, is controlled by both AOB and AOA, but the contributions of AOB and AOA to nitrification vary depending on environmental conditions [47]. Most reports show that biochar addition has a significant influence on the richness, diversity and community structures of AOB and AOA, but the conclusions are inconsistent for different soil alkalinities, parent materials, and salinization types [6]. This was also the case under biochar application conditions. Zhang et al. reported that straw biochar application significantly shifted the AOB community composition, and the abundance of *amoA*-AOB gene contributed to soil potential nitrification rates (PNR), whereas the abundance of *amoA*-AOA gene was almost not responsive to biochar addition [10]. Similarly, Xu et al. discovered that a significant response in *amoA*-AOB gene abundance, rather than *amoA*-AOA gene abundance, was observed under biochar application, although biochar addition significantly increased the diversity indices of AOB and AOA [48]. In a recent study, Li et al. concluded that biochar stimulated *amoA*-AOB gene abundance, which was significantly more abundant than *amoA*-AOA gene abundance, but the *amoA* activity showed a significant negative correlation with soil salinity and water-soluble carbon [49]. Most of the above findings were in accord with the present study in that AOB made a larger contribution to the nitrification rate and were more abundant than AOA. However, the present study showed that biochar suppressed *amoA*-AOB and *amoA*-AOA gene abundance. The explanation was that biochar addition decreased the frequency of functional microorganisms by increasing the diversity of AOB and AOA, and increased soil pH (Table 1), which exceeded the optimum value for *amoA* growth and activity [50].

The community structures of AOB and AOA showed distinct responses to biochar addition, with AOB being more susceptible than AOA to biochar. Most of the previous reports linked the functional changes with the abundance of comammox *Nitrospira*. Lin et al. reported that biochar addition increased the soil pH, diversity and abundance of *amoA* gene, and shifted the AOB community structure from *Nitrosospira*-dominated to *Nitrosomonas-dominated* [19]. Shi et al. discovered that *Nitrosospira* was the dominant genus in saline-alkali soil with a high NO_3_^−^-N content and salinity level, and biochar addition decreased the relative abundance of *Nitrosomonas* but increased that of *Nitrosovibrio* [16]. These findings were consistent with the present study, which confirmed that biochar addition decreased the relative abundance of *Nitrosospira* and *Nitrosomonas* but increased that of *Nitrosovibrio*. Li et al. reported that the reduced nitrification under biochar addition was mainly ascribed to the decrease in the abundance of comammox *Nitrospira*, which played a pivotal role in driving soil nitrification [51]. Bi et al. found that biochar addition shifted the community composition of AOB rather than AOA, with *Nitrosospira* Cluster 3a and Cluster 0 as the single predominant group of AOB [52]. More recently, Zhao et al. concluded that salinity gradients shaped the community composition and ecophysiology of comammox *Nitrospira*, which showed a clear response to a wide range of salinity levels [53]. This was consistent with [17] that the AOB community structure was more responsive to soil salinity dynamics induced by biochar application, and the biochar application rate affected the community composition of AOB and nitrification.

### 4.3. Causality among Soil Environmental Traits, Community Structures, amoA Gene Copies and Nitrification Rate under Biochar Addition

Genera *Nitrosospira*, *Nitrosomonas*, *Nitrososphaera* and *Nitrosopumilus* were dominant in community structure of ammonia-oxidizing microorganisms in the salt-affected irrigation-silting soil. This was in line with [54] that in a salt-affected alluvial delta area, the genus *Nitrosopumilus* dominated ammonia oxidization in vegetable soil and *Nitrosospira* dominated ammonia oxidization in wheat-maize rotation soil, whereas the dominant taxon was *Nitrosomonas* in paddy soil. The NR and numbers of *amoA*-AOB and *amoA*-AOA gene copies had significantly positive correlation with the relative abundance of *Nitrosospira*, and a negative correlation with the relative abundance of *Nitrosovibrio*. The relative abundance of genera *Nitrosomonas* and *Nitrososphaera* was negatively correlated with the numbers of *amoA*-AOB and *amoA*-AOA gene copies. These findings were consistent with Zhang et al., who reported that AOB rather than AOA dominated nitrification activity and that *Nitrosospira* Cluster 3-like AOB predominantly catalyzed bacterial ammonia oxidation [55]. In contrast, Xu et al. discovered that the genus *Nitrososphaera* played a critical role in the soil autotrophic nitrification activity of acidic upland soils, whereas nitrification activity was negatively correlated with the relative abundance of *Nitrosospira* [36]. This result was not unexpected as AOB dominated autotrophic nitrification in alkaline soil, but AOA were dominant in acidic soil. Recently, Hou et al. found that under biochar addition, the genus *Nitrosomonas* was the most abundant bacteria in microaggregates and positively correlated with the nitrification rate in acidic paddy soil [56]. This was also found by [57] in a saline aquaculture biofilm.

Nitrification, as a microbe-mediated process, was functionally associated with the abundance and community structures of AOB and AOA, which was more likely to result from the shifts in soil environmental traits induced by biochar addition [56]. In the present study, SEM captured the causal relationships among the soil properties, relative abundance of dominant taxa of AOB and AOA, and abundance of *amoA* gene on the nitrification rate. SOC and NO_3_^−^-N explained most of the structural variation in the AOB and AOA communities. Both SOC and soil initial NO_3_^−^-N content exhibited negative and positive indirect influences on NR. Evidence from [58] confirmed that the small-scale variation in ammonia oxidizers within saline sediments was dominated by *Nitrosomonas* and *amoA*-AOB abundance. It was also reported that combined biochar and urea amendment improved the relative abundance of *Nitrosomonas*, which dominated over *Nitrosospira* and nitrite-oxidizing bacteria (NOB) communities [59]. Moreover, the abundance of AOA showed a negative correlation with nitrification potential for the soil with a C:N ratio greater than 10 [60]. This partially explained the negative influence of *amoA*-AOA gene abundance on NR. In the present study, the potential mechanism of nitrification inhibition is that biochar addition improved the alpha diversity of AOB and AOA communities, shifted the community structure and decreased the relative abundance of dominant ammonia-oxidizers. This could be witnessed from the correlation among NR, *amoA* gene copies and Shannon index for AOB and AOA (Supplementary Figure S2). Moreover, soil microhabitat traits including high pH, C:N ratio, organic matter, soil salinity also contributed to nitrification inhibition. Hou et al. pointed out that the initial soil fertility status, which was closely related to basal nitrification, should be fully considered when using biochar to mediate nitrification [56].

## 5. Conclusions

Under N fertilization conditions, biochar addition inhibited the average nitrification rate and numbers of *amoA*-AOB and *amoA*-AOA gene copies in moderately salinized irrigation-silting soil, and the inhibitory effect increased with the biochar application rate. Biochar addition decreased the OTUs of both AOB and AOA, increased the alpha diversity of AOB but decreased that of AOA. Biochar addition and N fertilization shifted the AOB community structure from *Nitrosospira*-dominated to *Nitrosospira* and *Nitrosomonas-dominated* and changed the AOA community from *Nitrososphaera*-dominated to *Nitrososphaera* and *Nitrosopumilus*-dominated. For the AOB community, biochar addition decreased the relative abundance of *Nitrosospira* and *Nitrosomonas*, but increased that of *Nitrosovibrio*, *Unclassified_k_norank_d_Bacteria* and *Norank_p_ammonia_ oxidising_bacteria_ensemble* under N fertilization. For the AOA community, biochar addition enhanced the relative abundance of *Nitrososphaera*, but decreased that of *Nitrosopumilus* under N fertilization. Soil microhabitat traits including SOC, pH and NO_3_^−^-N explained 87.1% of the total variation in the AOB community, and a total of 78.1% variation in the AOA community was explanatory by soil pH and SOC. Results of structural equation models (SEMs) showed that SOC had indirect influence on NR through *Nitrosovibrio*, *Norank_c_environmental_samples_p_Crenarchaeota* and *amoA*-AOB and *amoA*-AOA gene abundance, and NO_3_^−^-N had indirect influence on NR through *Nitrosomonas*, *Norank_c_environmental_samples_ p_Crenarchaeota* and *amoA*-AOB and *amoA*-AOA gene abundance. Our conclusion is that biochar addition inhibits nitrification by improving the community diversity of AOB, shifting the community structures of AOB and AOA, and reducing the relative abundance of dominant functional ammonia-oxidizers. Soil microhabitat traits and unclassified ammonia-oxidizing microorganism also play an important role in nitrification inhibition, which still needs further efforts to validate using long-term observation experiments.

## Figures and Tables

**Table 1:** Basic soil properties for all the treatments (mean ± standard deviation), and the one-way ANOVA results with the least significant difference (LSD).

Treatments	EC_1:5_ (dS⋅m^−1^)	pH	CEC (cmol⋅kg^−1^)	SOC (g⋅kg^−1^)	TN (g⋅kg^−1^)	NH_4_^+^-N (mg⋅kg^−1^)	NO_3_^-^-N (mg⋅kg^−1^)	AP (mg⋅kg^−1^)
CK	1.36 ± 0.13 a	8.24 ± 0.04 c	14.03 ± 0.23 c	11.51 ± 0.21 c	0.60 ± 0.08 b	14.98 ± 1.64 c	35.72 ± 2.89 c	21.67 ± 2.67 bc
N	1.42 ± 0.20 a	8.18 ± 0.03 c	14.55 ± 0.43 bc	13.43 ± 0.63 b	0.69 ± 0.14 ab	20.53 ± 1.21 b	51.29 ± 3.19 ab	19.28 ± 1.91 c
NB1	1.31 ± 0.16 a	8.32 ± 0.07 b	15.12 ± 0.49 b	13.82 ± 0.95 ab	0.72 ± 0.15 a	22.27 ± 1.27 ab	50.11 ± 3.20 ab	23.10 ± 2.41 b
NB2	1.24 ± 0.10 a	8.40 ± 0.03 a	17.45 ± 1.08 a	14.83 ± 0.27 a	0.69 ± 0.07 ab	20.65 ± 2.56 b	53.51 ± 2.17 a	26.49 ± 1.55 ab
NB3	1.21 ± 0.02 a	8.41 ± 0.02 a	17.78 ± 0.23 a	14.43 ± 0.64 a	0.67 ± 0.16 ab	24.40 ± 2.11 a	46.43 ± 1.58 ab	28.38 ± 3.50 a

EC_1:5_: electrical conductivity of 1:5 soil:water extract; CEC: cation exchange capacity; SOC: soil organic carbon; TN: total nitrogen; NH_4_^+^-N: ammonium nitrogen content; NO_3_^−^-N: nitrate nitrogen content; AP: available potassium. Different lowercase letters indicate significance at *p* ≤ 0.05.

**Table 2:** Nitrification rate and the numbers of *amoA*-AOB and *amoA*-AOA gene copies for all the treatments, plus the one-way ANOVA results with the least significant difference (LSD).

Treatments	Nitrification Rate (NR) (mg⋅kg^−1^⋅d^−1^)	*amoA*-AOB Gene Copies (×10^7^ g^−1^ Soil)	*amoA*-AOA Gene Copies (×10^6^ g^−1^ Soil)
CK	28.15 ± 6.13 a	8.21 ± 0.54 a	3.71 ± 0.49 a
N	27.64 ± 5.72 a	4.61 ± 0.64 b	2.12 ± 0.20 b
NB1	21.80 ± 4.01 ab	3.52 ± 0.63 bc	1.84 ± 0.36 b
NB2	15.74 ± 4.42 b	2.97 ± 0.81 c	0.96 ± 0.05 c
NB3	11.67 ± 7.58 b	2.50 ± 0.43 c	0.71 ± 0.09 c

Different lower case letters indicate significance at *p* ≤ 0.05.

**Table 3:** Richness and diversity indexes of AOB and AOA, plus the statistical comparison of community indexes among all treatments using the one-way ANOVA with the least significant difference (LSD).

Treatment	AOB					AOA				
	OTUs	Chao1	Shannon	Simpson	Coverage (%)	OTUs	Chao1	Shannon	Simpson	Coverage (%)
CK	22.33 ± 1.53c	23.83 ± 2.75 b	1.93 ± 0.11 c	0.18 ± 0.04 a	99.97 ± 0.01 a	30.33 ± 4.04 c	34.22 ± 4.83 a	1.28 ± 0.04 d	0.40 ± 0.01 a	99.95 ± 0.02 a
N	32.33 ± 2.08 a	33.33 ± 0.58 a	2.14 ± 0.06 b	0.16 ± 0.01 a	99.98 ± 0.02 a	38.33 ± 2.08 a	41.00 ± 4.00 a	2.15 ± 0.07 a	0.17 ± 0.02 d	99.97 ± 0.01 a
N B1	31.33 ± 2.89 ab	31.79 ± 3.29 a	2.11 ± 0.10 b	0.17 ± 0.02 a	99.99 ± 0.02 a	36.33 ± 3.21 ab	40.67 ± 4.30 a	1.76 ± 0.07 c	0.26 ± 0.01 b	99.96 ± 0.02 a
N B2	30.67 ± 2.52 ab	31.00 ± 2.78 a	2.28 ± 0.10 b	0.20 ± 0.03 a	99.99 ± 0.01 a	35.33 ± 3.22 ab	40.83 ± 10.56 a	1.92 ± 0.06 b	0.21 ± 0.01 c	99.96 ± 0.02 a
N B3	29.33 ± 0.58 b	33.67 ± 5.03 a	2.47 ± 0.10 a	0.11 ± 0.01 b	99.97 ± 0.03 a	35.00 ± 1.00 b	39.28 ± 1.84 a	1.98 ± 0.04 b	0.20 ± 0.01 c	99.95 ± 0.02 a

Different lowercase letters indicate a significant difference at *p* < 0.05.

**Table 4:** Relative abundance (%) of predominant taxa for AOB and AOA at the order level across all the treatments.

	Taxa	Treatment				
		CK	N	NB1	NB2	NB3
AOB	*Nitrosomonadales*	95.44 ± 1.33 a	81.59 ± 0.82 ab	73.26 ± 9.68 bc	63.08 ± 16.96 c	58.87 ± 3.45 c
	*Unclassified_k_norank_d_Bacteria*	1.07 ± 1.04 c	16.35 ± 0.87 b	23.27 ± 8.83 ab	30.94 ± 11.51 a	31.10 ± 3.16 a
	*Norank_p_ammonia_oxidising_bacteria_ensemble*	3.32 ± 1.92 b	1.42 ± 0.31 c	2.97 ± 0.52 b	5.40 ± 2.39 ab	9.90 ± 1.62 a
AOA	*Nitrososphaerales*	37.95 ± 1.47 b	43.15 ± 10.45 b	58.76 ± 11.75 a	60.23 ± 7.82 a	51.02 ± 4.17 ab
	*Nitrosopumilales*	-	29.37 ± 6.56 a	26.79 ± 14.00 ab	14.41 ± 11.43 ab	12.90 ± 6.85 b
	*Norank_c_environmental_samples_p_Crenarchaeota*	57.63 ± 1.00 a	12.10 ± 3.16 b	2.45 ± 0.80 d	3.85 ± 1.65 d	7.84 ± 1.54 c
	*Unclassified_k_norank_d_Archaea*	4.24 ± 0.55 c	14.39 ± 2.05 b	11.78 ± 1.67 bc	20.87 ± 11.30 ab	27.65 ± 6.20 a

Different letters indicate significant differences among different treatments for each taxonomic order based on least significant difference (*p* ≤ 0.05).

**Table 5:** Spearman’s rank correlations between the relative abundances of dominant genera of AOB and AOA, and nitrification rate, gene copies of *amoA*-AOB and *amoA*-AOA, and the soil basic properties (*n* = 15).

	Genus	NR	*amoA*-AOB	*amoA*-AOA	EC_1:5_	pH	CEC	SOC	TN	NH_4_^+^-N	NO_3_^−^-N	AP
AOB	*Nitrosospira*	0.622 *	0.944 **	0.938 **	0.387	−0.620 *	−0.737 **	−0.859 **	−0.489	−0.784 **	−0.712 **	−0.534 *
	*Nitrosomonas*	−0.250	−0.67 **	−0.564 *	−0.196	0.188	0.201	0.562 *	0.505 *	0.759 **	0.781 **	0.075
	*Unclassified_o_Nitrosomonadales*	−0.301	−0.564 *	−0.657 **	−0.101	0.390	0.695 **	0.662 **	0.366	0.341	0.385	0.488
	*Nitrosovibrio*	−0.640 **	−0.505 *	−0.623 **	−0.398	0.575 *	0.736 **	0.394	−0.149	0.504 *	0.059	0.682 **
	*Unclassified_k_norank_d_Bacteria*	−0.635 **	−0.864 **	−0.864 **	−0.333	0.641 **	0.700 **	0.779 **	0.395	0.672 **	0.607 *	0.525 *
	*Norank_p_ammonia_oxidising_bacteria_ensemble*	−0.549 *	−0.379	−0.437	−0.565 *	0.572 *	0.536 *	0.285	−0.030	0.156	−0.106	0.507 *
AOA	*Nitrososphaera*	−0.387	−0.599 *	−0.592 *	−0.287	0.515 *	0.464	0.631 **	0.193	0.538 *	0.558 *	0.395
	*Nitrosopumilus*	0.048	−0.434	−0.283	0.171	−0.156	0.014	0.368	0.684 **	0.415	0.598 *	−0.162
	*Norank_c_environmental_samples_p_Crenarchaeota*	0.502 *	0.936 **	0.851 **	0.225	−0.429	−0.581 *	−0.853 **	−0.647 **	−0.792 **	−0.864 **	−0.339
	*Unclassified_k_norank_d_Archaea*	−0.687 **	−0.749 **	−0.785 **	−0.411	0.581 *	0.728 **	0.612 *	0.233	0.518 *	0.394	0.532 *

* indicates significance at *p* ≤ 0.05; ** indicates significance at *p* ≤ 0.01.

## Data Availability

The data and results of this study are available upon reasonable request. Please contact the main author of this publication.

## Supplementary Materials

The following supporting information can be downloaded at: https://www.mdpi.com/article/10.3390/microorganisms10020436/s1, Figure S1: Temporal dynamics of nitrification rate at different times across the incubation period, plus one-way analysis of variance (ANOVA) with the least significant difference (LSD). ** indicates significance at *p* ≤ 0.01; Figure S2: Relationships among nitrification rate, *amoA* gene copies and Shannon index for AOB and AOA communities; Table S1: The amplification primer, sequence and reaction condition of quantitative PCR for *amoA*-AOB and *amoA*-AOA genes.
