# Supplementary material for: Biochar Addition Inhibits Nitrification by Shifting Community Structure of Ammonia-Oxidizing Microorganisms in Salt-Affected Irrigation-Silting Soil
Source: Microorganisms. 2022 Feb 14;10(2):436. doi: 10.3390/microorganisms10020436 (PMC8878283; doi:10.3390/microorganisms10020436)
Supplement: Supplementary file 1 [file microorganisms-10-00436-s001.zip › microorganisms-1606929-supplementary.pdf]

Table S1 The amplification primer, sequence and reaction condition of quantitative PCR for *amoA*-AOB and *amoA*-AOA genes

Target gene	Primer	Sequence (5'−3')	Thermal cycling	Reference
amoA-AOB	*amoA*-1F	GGGGTTTCTACTGGTGGT	94 °C − 10 min; 94 °C – 30 s, 40 cycles;	Hussain et al., 2011
	amoA-2R	CCCCTCKGSAAAGCCTTCTTC	53 °C – 30 s; 72 °C – 45 s	
amoA-AOA	Arch-*amoAF*	STAATGGTCTGGCTTAGACG	94 °C − 10 min; 94 °C – 30 s, 40 cycles;	Francis et al., 2005
	Arch-*amoAR*	GCGGCCATCCATCTGTATGT	53 °C – 30 s; 72 °C – 45 s	

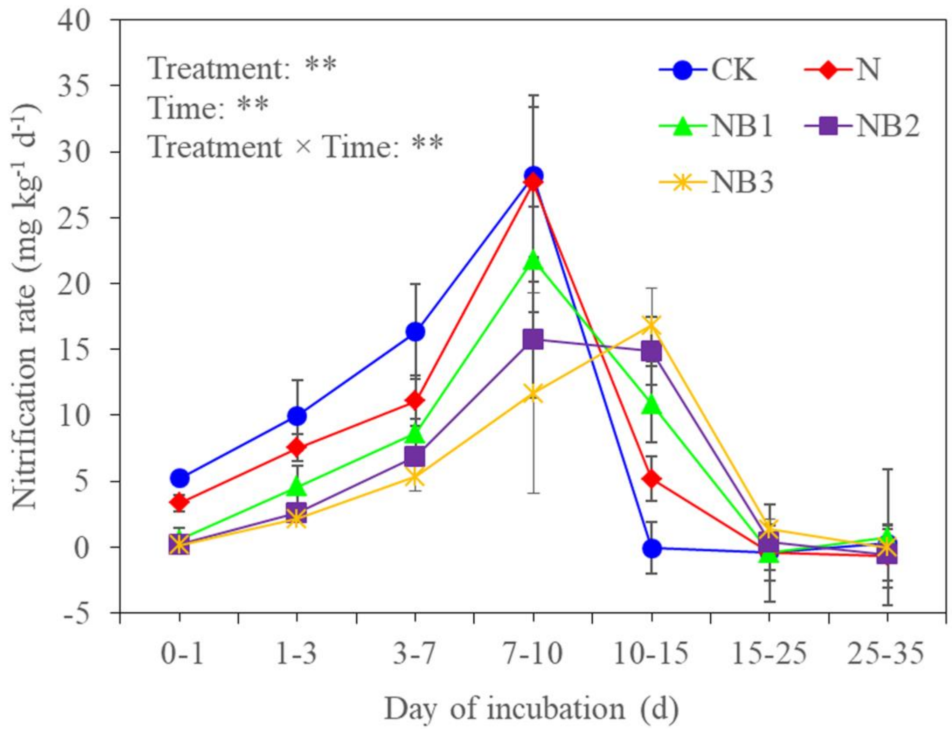

Figure S1 Temporal dynamics of nitrification rate at different times across the incubation period, plus one-way analysis of variance (ANOVA) with the least significant difference (LSD). ** indicates significance at $p \leqslant 0.01$

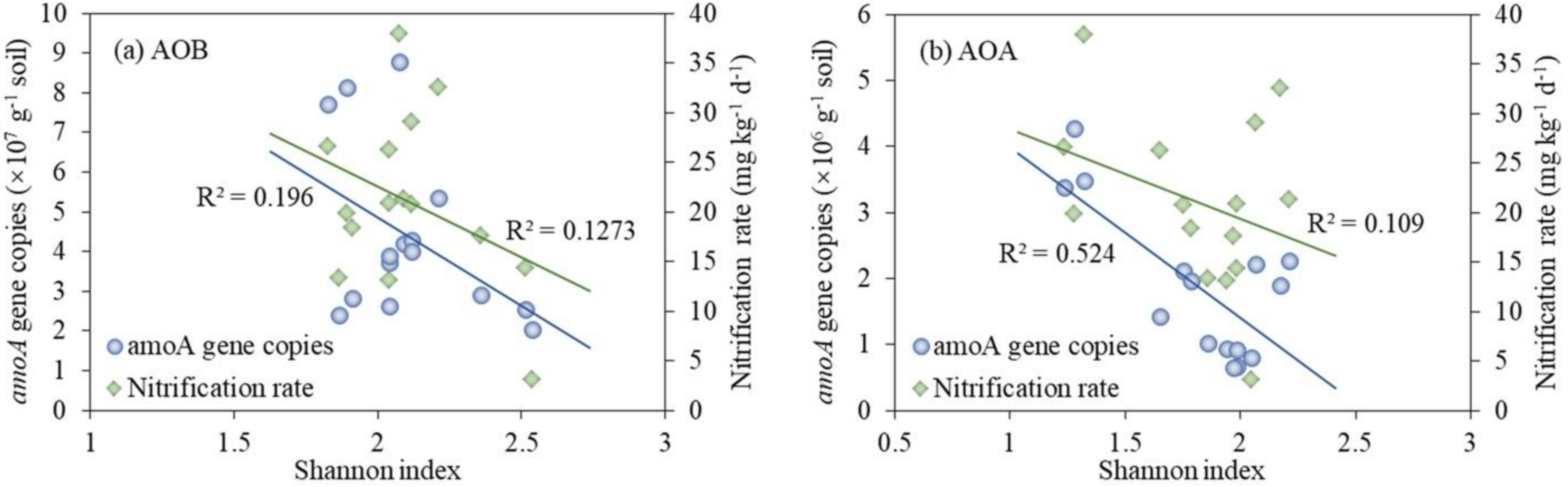

Figure S2 Relationships among nitrification rate, *amoA* gene copies and Shannon index for AOB and AOA communities
